# The inhibition of motor contagion induced by action observation

**DOI:** 10.1371/journal.pone.0205725

**Published:** 2018-10-17

**Authors:** Tatsuya Takeuchi, Sachi Ikudome, Satoshi Unenaka, Yasumitsu Ishii, Shiro Mori, David L. Mann, Hiroki Nakamoto

**Affiliations:** 1 Faculty of Physical Education, National Institute of Fitness and Sports in Kanoya, Kagoshima, Japan; 2 Department of Sport Education, School of Lifelong Sport, Hokusho Universuty, Hokkaido, Japan; 3 Department of Sports Science, Japan Institute of Sports Sciences, Tokyo, Japan; 4 Faculty of Human Movement Sciences, Vrije Universiteit Amsterdam, Amsterdam Movement Sciences and Institute Brain and Behavior Amsterdam, Amsterdam, The Netherlands; Universita degli Studi di Verona, ITALY

## Abstract

In sports, success and failure are believed to be contagious. Yet it is unclear what might cause contagion. This study investigated whether motor contagion is associated with the active observation of the kinematic actions of others. In Experiment 1, six skilled hammer throwers threw a hammer after watching a video of a model throwing toward the left, center, or right. The video included two types of action kinematics which resulted in throw directions that were either easy or difficult to predict based on the model’s kinematics. In Experiment 2, the athletes threw hammers after watching the same stimuli as Experiment 1, but while engaging in one of two types of focus (self-focus or non-self-focus) to determine whether motor contagion could be diminished. Results demonstrated that the direction of each participant’s throw was more influenced by the videos that contained easy action kinematics, supporting a critical role for the meaningfulness of the link between an action and its outcome in producing motor contagion. Motion analysis revealed that motor contagion was not likely to be a result of the observer imitating the model’s action kinematics. The contagion observed in Experiment 1 disappeared when participants engaged in self-focus. These results suggest that motor contagion is influenced by the predictability of an action outcome when observing an action, and that motor contagion can be inhibited through self-focus when observing.

## Introduction

In various sport settings, success and failure are contagious. For example, batting averages in baseball increase when a batter follows a teammate who scored a hit, but decrease following a batter who was out [1,2]. Ikegami and Ganesh [3] discussed an interview with Ichiro Suzuki, a star hitter who established a number of batting records in US Major League Baseball, in which Ichiro told them that “[h]e refrains from closely watching poor batters on his team before going out to bat because it affects his own batting performance.” Although many factors may be related to this phenomenon of *contagion* in sports (e.g., emotional [4], mood [5], and social contagion [6]), some researchers have recently focused on *motor contagion*—when an actor experiences implicit effects on their own actions on the basis of the actions of others—as one influential factor of the contagion phenomenon [3,7]

Gray and Beilock [7] experimentally investigated motor contagion among baseball batters to test the belief that “hitting is contagious.” In their experiment, experienced and less-experienced baseball batters were asked to hit virtual baseballs toward a center direction after having observed one of three types of action-inducing stimuli: an action prompt (a ball traveling from the home plate into the left, right, or center field), an outcome prompt (a ball resting in either the left, right, or center field), or a verbal prompt (text showing the word “left,” “right,” or “center”). They found that the direction of the prompt significantly influenced the direction of the experienced batters’ hits in both the action and outcome prompt conditions, but not for the verbal prompt or when less experienced batters were hitting. If the inducing stimulus in the action prompt condition was, for instance, a ball traveling to the left field, then experienced batters hit the baseball more to the left, even when they were instructed to hit toward the center. Further, successful outcomes (i.e., making hits) were more likely after observing the action prompt. These results demonstrate that the mere observation of an action outcome induces a contagion effect in observers and may help to support the belief that “hitting is contagious” in baseball [1,2].

Note that when contagion was present in the Gray and Beilock [7] study, observers did not see the kinematic action of the model hitting a ball. Instead, participants saw the ball either moving in one direction, or saw it stationary in its final position. Research outside of sports has shown that motor contagion can be induced by observing the action kinematics of others, a phenomenon often referred to as *automatic imitation* [8–11]. This is the tendency to copy observed actions even when they are not relevant to the task at hand [12]. Substantial behavioral evidence for automatic imitation has been demonstrated for reaction time tasks [8,9,11], object grasping [13], cyclical movement tasks [14], and even when playing rock-paper-scissors [15]. These findings indicate that mere observation induces the unconscious imitation of a model’s action, irrespective of one’s own goal [16]. Thus, it could be that motor contagion may also occur in sport by inducing automatic imitation when observing the action kinematics of others.

One experiment has already shown that the observation of another person’s kinematics may influence performance in sports. Ikegami and Ganesh [3] asked expert dart throwers to watch videos of novice throwers, with the dart flight-trajectory and dart board masked so that only information about the throwing kinematics was available. Participants were asked to predict the location in which the novice’s throws would have hit the dart board. As the experiment progressed, experts substantially improved their ability to predict the outcome of the throws. Interestingly, this improvement led to a deterioration in the experts' own throwing performance. One possible explanation for this change is that the observer’s own kinematics might have changed by observing another person produce a similar action. Alternatively, action observation could result in motor contagion without necessarily changing the observer’s own kinematics. In support, previous studies have found in some cases that observers imitate the *intention or goal* of the observed action, rather than the action kinematics of the observed action [17,18]. Therefore, it remains unclear whether motor contagion is induced by *kinematic* imitation after observing an action. To date, movement kinematics have not been analyzed to resolve this question.

It has been suggested that the motor contagion which results from action observation may be more likely when there is a strong link between the observed action and its outcome, because motor contagion is enhanced when the observer is more accurate in anticipating the outcome they are predicting [3]. In a similar vein, Gray and Beilock [7] stated that motor contagion among baseball players was more common among experienced players than among less-experienced players because more experienced players are increasingly likely to be able to associate the observed outcome with the motor action that was required to achieve that outcome. Motor contagion and related phenomena are often explained by common coding theory, which assumes that common representations underpin the observation and execution of the same motor action [19,20]. This theory suggests that action observation *automatically* activates one’s own motor system as if internally simulating a perceived action (for a review, [21]). In addition, it has been reported that expert athletes who have superior prediction abilities activate this motor system when they predict the outcome of an action performed by others [22–25], especially when making successful predictions [26]. Therefore, it seems reasonable to expect that motor contagion will be more likely to be found when observers can predict the action outcome on the basis of the observed action kinematics. Thus, the purpose of this first study was to test whether the ability to predict the outcome of an observed action would influence the degree of motor contagion in sport (Experiment 1).

## Experiment 1

In Experiment 1, highly skilled hammer throwers were required to throw a hammer toward the center of a field after they had watched videos of a model throwing to one of three locations. The videos ended at the moment the hammer was released to ensure that only the action kinematics of the model were seen (without the hammer trajectory and outcome). Moreover, to manipulate the strength of the association between the action and the outcome, we presented two types of videos that differed in the degree to which the direction of the throw could be predicted on the basis of the model’s action kinematics: a trunk strategy in which the prediction of the direction of the throw was harder (hard prediction, HP) and a step strategy in which the prediction of direction was easier (easy prediction, EP) (see Apparatus and stimuli in detail). Given that a link between the action and outcome appears to be necessary for motor contagion to occur, it was hypothesized that the participant’s direction of throw would be more susceptible to motor contagion in the EP condition than it would in the HP condition. Furthermore, we recorded movement kinematics during the hammer throw to identify whether any motor contagion could be explained on the basis of kinematic imitation.

### Methods

#### Participants

Six (3 male and 3 female) highly skilled varsity hammer throwers belonging to a college track and field team took part in Experiment 1. Their mean age was 20.8 ± 1.2 yrs and their mean years of competitive playing experience was 5.8 ± 1.2 yrs. All were national-level players in Japan, and each generally spent 16 hours per week practicing. The experiment was undertaken with the written informed consent of each participant. This study was approved by the Ethics Committee of the National Institute of Fitness and Sports in Kanoya (approval number 3–8) and was consistent with the institutional ethical requirements for human experimentation in accordance with the Declaration of Helsinki.

#### Apparatus and stimuli

**Fig 1:**
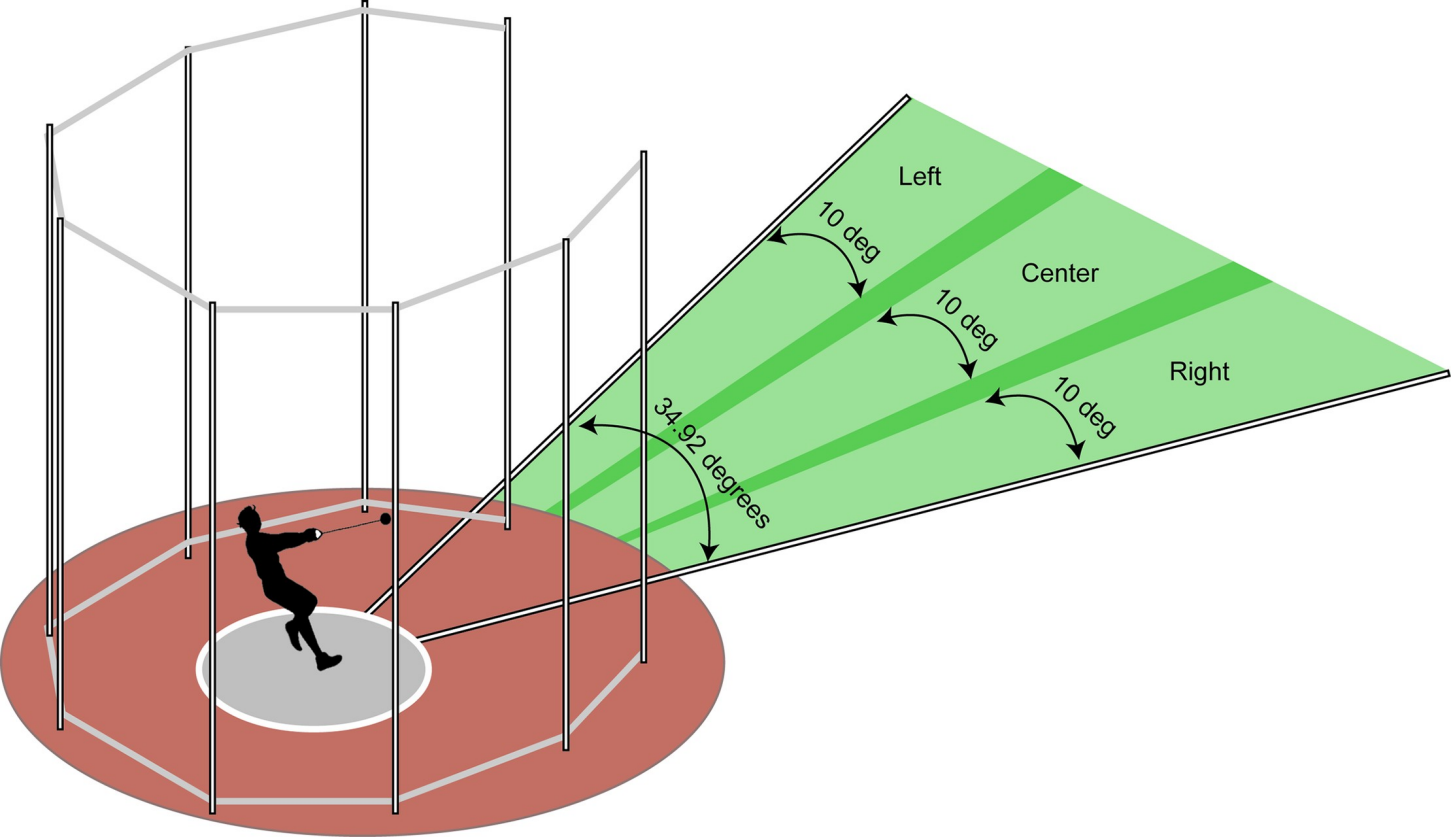
The landing field where are present the areas (right, center, and left) considered as valid in video recording and motion capture of model hammer throwing.

Participants threw a hammer on an outdoor hammer field which complied with international standards. All visual stimuli were presented on a large 76.9 × 121.8 cm monitor (TH-P50G1, Panasonic) located 350 cm away from the hammer throwing circle. Stimuli consisted of a model who threw in one of three directions (left, center, and right), and with one of two types of action kinematics (HP and EP). To create the stimulus videos, we recorded a right-handed national-level athlete with 7 years of experience, using a digital video camera (HDR-CX560V, Sony) at 60 Hz. A 4.00 kg hammer recognized by the National Athletic Federation was used. To create the stimuli, the model threw the hammer while aiming towards one of the three predefined directions: the center direction was defined as any throw that landed within the central ten degrees of the field; the left direction was within ten degrees inside of the left foul line; and the right direction was within ten degrees inside of the right foul line (Fig 1). Any other throws were excluded from the test stimulus.

**Fig 2:**
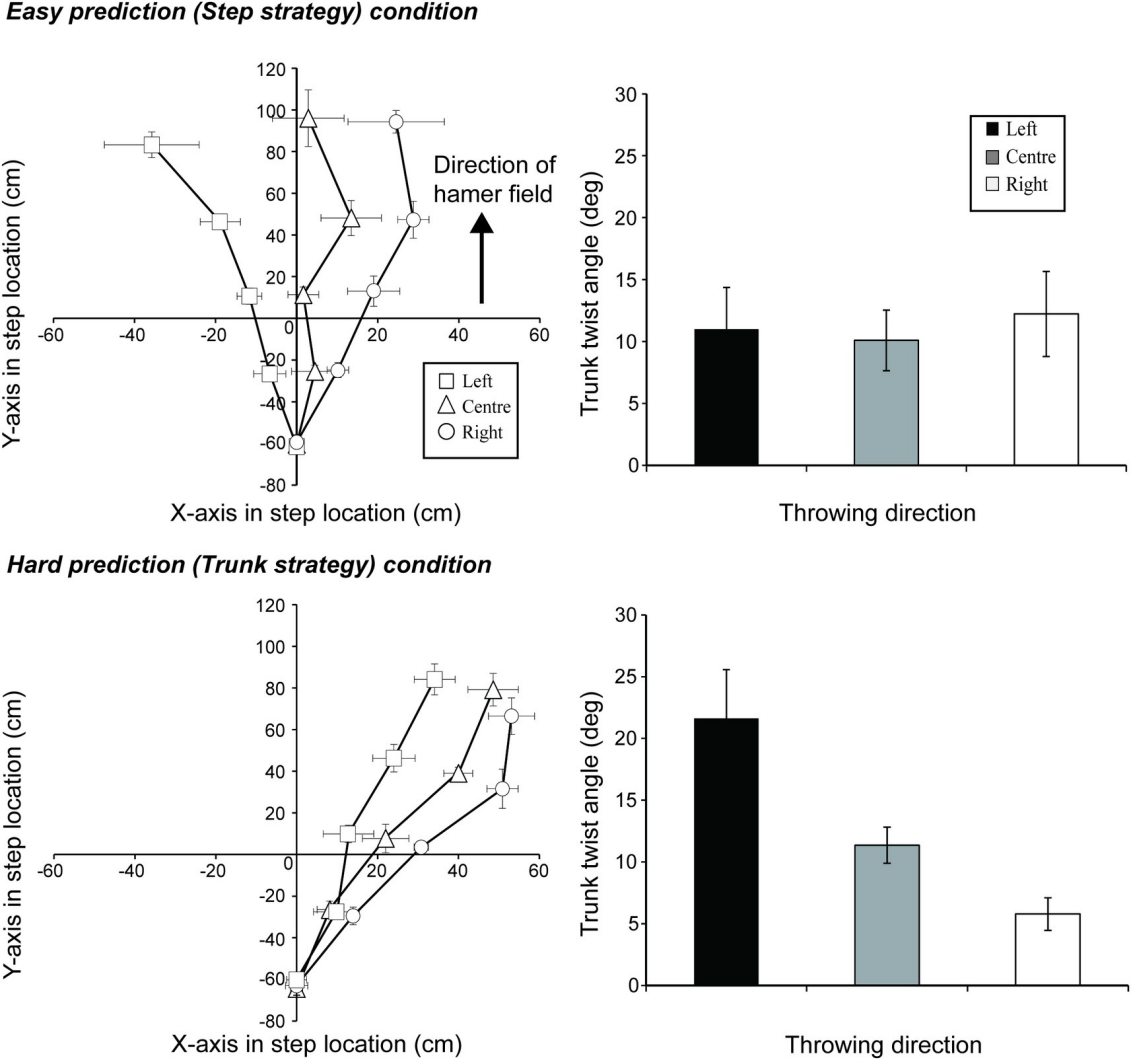
Model throwing kinematics for step location (left panel) and trunk twisting angle (right panel) used in the hard-outcome prediction (upper panel) and easy-outcome prediction (lower panel) video conditions.

To manipulate the ease with which the direction of throw could be predicted, the model threw the hammer using one of two different techniques. In the HP condition, the model threw the hammer toward the instructed locations by regulating the trunk twist angle (*trunk strategy*; see lower panel in Fig 2). The trunk twist angle was defined as the angle between the line joining the right and left acromion, and the line joining the right and left anterior superior iliac spine. The larger the twist angle, the more the hammer went toward left field, and the smaller the twist angle, the more the hammer went toward right field. In this condition, the model was instructed to keep their pivot-step location as similar as possible across the different directions of throw. Pivot-step location was defined as the position of the left foot that became the pivot leg when turning. In the EP condition, the model threw the hammer toward the intended direction by regulating only the pivot-step location (*step strategy*; see upper panel in Fig 2). In this condition, the model was asked to maintain the same trunk angle across the directions of throw. The model continued to throw the hammer until he or she had performed ten successful throws toward each of the three directions using each of the two different techniques.

The recorded videos were edited using video editing software (Premiere Elements 2.0, Adobe). Five of the ten videos were chosen for each of the three directions and two kinematic conditions. The trials that were selected were those where the kinematics most closely matched the desired technique in that condition (see below). A warning cross was added to videos before each trial (duration 2000 ms) to alert the participant that the trial would start, followed by the footage of the kinematic action of the hammer throw (about 900 ms), and then white noise for 2000 ms.

To analyze the kinematics of both the model and participants, kinematic data were recorded with an eleven-camera digital three-dimensional motion analysis system (Motion Analysis Corporation, Santa Rosa, CA) working at a frame rate of 600 Hz. Calibration was conducted using the wand calibration method. The root mean square of positional error was less than 1.0 mm in three-dimensional space. Reflective markers were attached to 29 locations on the hammer and the model or participants’ cephalic part, upper body, and lower body. Twenty-seven reflective markers (1.25 cm in diameter) were attached to their anatomical landmarks, including 23 markers placed bilaterally on the frontal, parietal, and occipital part of the head, acromion, lateral epicondyle of the humerus, radial styloid and ulnar styloid, anterior superior iliac spine, greater trochanter, lateral and medial condyle of the tibia, and lateral and medial malleolus of the tibia. Four markers were placed bilaterally on the toe and heel of the throwing shoes. Moreover, to identify the moment of hammer release, two reflective markers were attached to the handle of the hammer. The three-dimensional coordinate system was defined with the y-axis as the straight direction of throw, the z-axis as the vertical axis, and the x-axis as the horizontal axis located toward the right side of the hammer circle. The moment of hammer release was defined as the moment when the reflective marker on the hammer handle deviated from the markers on the hands.

To verify the difference in the predictability of the two different throwing techniques, we conducted a preliminary outcome prediction task before Experiment 1. In this task, at least two days before starting the experiment proper, the six skilled hammer throwers observed the video clips that occluded at hammer release. After each clip, participants were required to predict where the hammer would have landed on the field (left, center, or right). Participants observed a total of 60 clips, consisting of 30 clips for each of the HP and EP conditions (10 clips for each of the three directions). The order of presentation of the direction of throw for each participant was randomized, with the order of EP presentation and HP presentation counterbalanced across participants. Feedback on outcomes was not given. A *t*-test between the HP and EP conditions demonstrated that the mean prediction accuracy was significantly higher in the EP than it was in the HP condition (HP vs. EP = 49 vs. 87%, *t*(5) = 8.50, p < .01) (S1 Table), verifying the difference in predictability between the two conditions.

#### Procedure

In Experiment 1, participants first completed the warm-up that they would typically perform before commencing practice. Next, to measure each participant’s normal throwing direction and movement kinematics, participants performed 15 hammer throws before observing any model videos (*normal* condition). Following the 15 normal trials, participants performed 15 hammer throws for each of the EP and HP stimulus conditions in a blocked fashion (all EP or all HP trials first, with the order of EP and HP presentation counterbalanced across participants). In both conditions, participants threw the hammer one time directly after observing five repetitions of the same video. In total, there were 15 unique videos in each of the EP and HP conditions (5 videos for each of the three directions). The order of presentation of the direction of throw was randomized within each EP and HP block. Thus, participants threw the hammer 45 times in total. We chose this number to avoid the influence of fatigue on throw direction. Moreover, participants were given a short break following each throw. In every condition, participants were instructed to throw the hammer toward the center of the hammer field, irrespective of what they had seen in the videos.

#### Measurements and data analysis

To clarify whether the direction of throw was affected by observing the videos, we used a digital protractor to calculate the transverse angle of throw direction based on where the hammer landed. A throw directly toward the center was defined as 0 degrees. The angle for any throw directed away from the center was derived by calculating the angular deviation of the landing location away from the center line. Positive values indicated a hammer falling to the right of center. Further, we subtracted the values of the average angle in the normal condition from the angle in the EP and HP conditions (defined as subtracted value) to determine the effect of observation on the throwing direction during performance, irrespective of individual differences in their ‘normal’ throwing direction. The mean subtracted throwing directions were analyzed using a 3 (Direction: left, center, and right stimuli) × 2 (Predictability: EP and HP) analysis of variance (ANOVA) with repeated measures on both factors.

Second, to clarify the cause of motor contagion (i.e., kinematic imitation or not), we calculated participants’ step locations and trunk twist angles during the hammer throws using the motion kinematic data. The definitions of trunk twist angle and step location were identical to those used for the model. To compare these values across conditions, we calculated from time-series data the step location and trunk twist angle at the moment of hammer release. The reason for considering only the moment of hammer release is that the difference in the trunk twist angle and step locations progressively increases as the moment of hammer release nears, with the maximum difference occurring at the moment of release. Therefore, we expected that any difference in the participant’s kinematics across conditions would be maximized at the moment of hammer release. Values for the step location (x-axis) and trunk twist angle in the HP and EP conditions were subtracted from those in the normal condition to test for changes as a result of the direction and predictability of the observed throw. The means of these subtracted values were analyzed using a 3 (Direction: left, center, and right stimuli) × 2 (Predictability: EP and HP) ANOVA. Although it is possible that step location and trunk twist angle could co-vary, Fig 2 shows that these factors can be individually manipulated largely independent of each other. This was particularly the case for the trunk twist angle: it was possible in the EP condition to produce large differences in step location without altering the trunk twist angle. This result supports the idea that there is a degree of degeneracy in the way that movements can be produced in the hammer throw, and in particular that the trunk angle and step locations can be manipulated and analyzed independently. Therefore, we considered the two measures independently in the analysis. Effect sizes and their 95% confidential intervals are reported as eta squared (η^2^). The effect sizes of 0.01, 0.06, and 0.14 were termed small, medium, and large, respectively. Also, observed power are reported, and post-hoc mean comparisons were performed using the Bonferroni test.

### Results and discussion

#### Direction of throw

**Fig 3:**
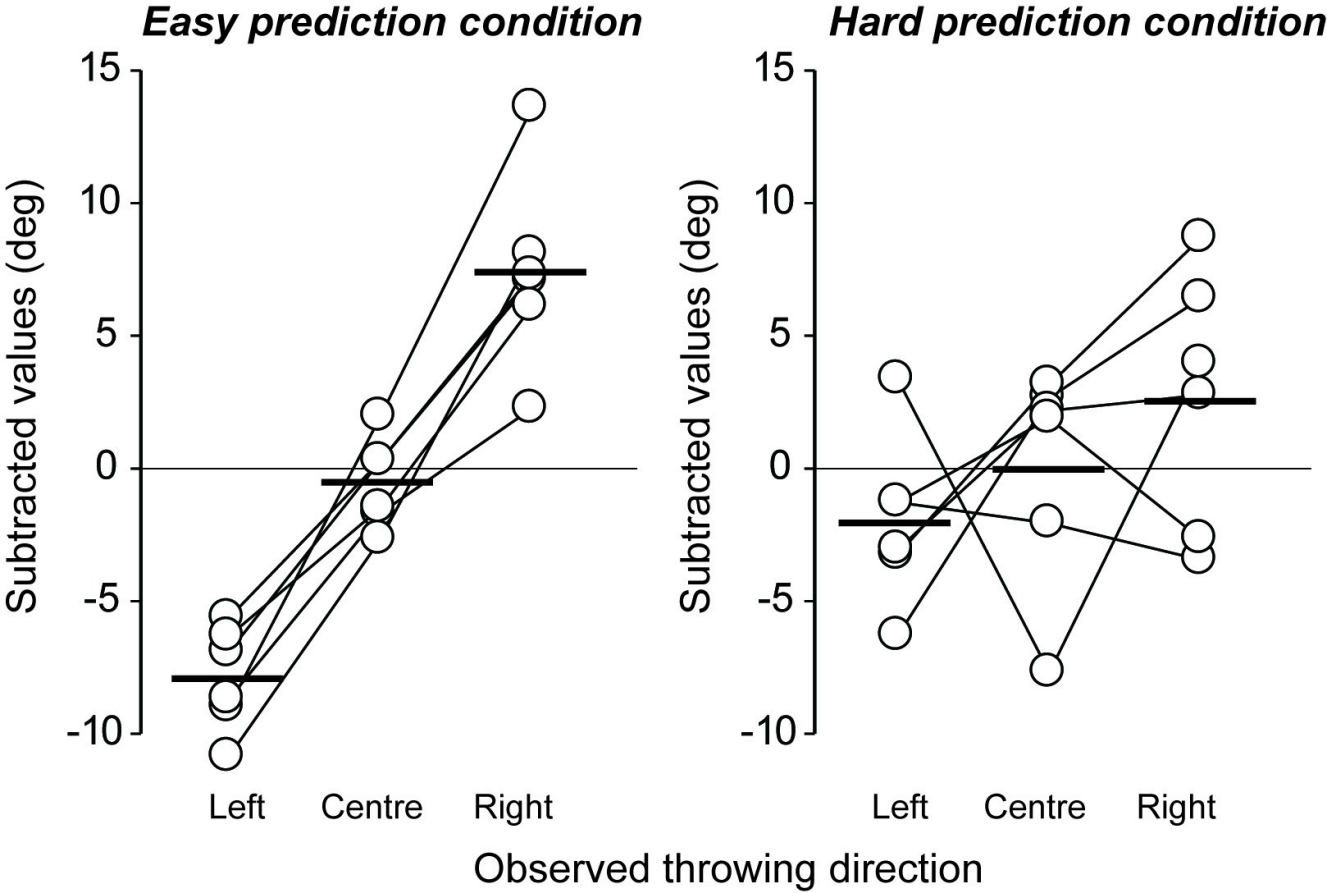
Individual data of hammer throwing direction after observing the model’s throwing kinematics directed to the left, center, and right. The data indicate subtracted values of transverse angle under the EP and HP stimulus conditions from the transverse angle under the normal condition, to illuminate individual differences in normal throwing direction. In the EP condition (left panel), the model’s throwing direction could be easily predicted from the model’s throwing kinematics. In the HP condition (right panel), it was difficult to predict the model’s throwing direction by observing the model’s throwing kinematics. Black thick bars indicate average values.

We hypothesized that if motor contagion relies on the accurate prediction of an action outcome, then motor contagion would be greater when viewing the EP stimuli where the link between the kinematics and action outcome is easier to predict. Fig 3 shows the direction of throw in the EP and HP conditions subtracted from that in the normal condition (see also, S1 Table). The findings revealed that motor contagion was indeed greater in the EP condition than it was in the HP condition. ANOVA testing revealed a significant main effect with large effect size for the direction of the observed throw (*F*(2, 10) = 20.47, *p* < .01, η^2^ = .53 [95% CI = 0.35–0.76], observed power = .99) and an interaction with large effect size between the direction of throw and predictability of outcome (*F*(2, 10) = 7.06, *p* < .05, η^2^ = .16 [95% CI = 0.04–0.33], observed power = .83; main effect of predictability, *F*(1,5) = 0.62, *p* = .46, η^2^ = .00 [95% CI = .00 - .02], observed power = .09). In the EP condition, there were significant differences in the direction of throw when comparing each of the left-center, left- right, and center-right stimulus conditions (*p*s < .01). In the HP condition, however, there was no difference between any of the different directions of throw. These results confirm that motor contagion is more likely to be induced when observing actions where the outcome of that action can be reliably predicted on the basis of the observed kinematics.

#### Does action observation change the observer’s movement kinematics?

**Fig 4:**
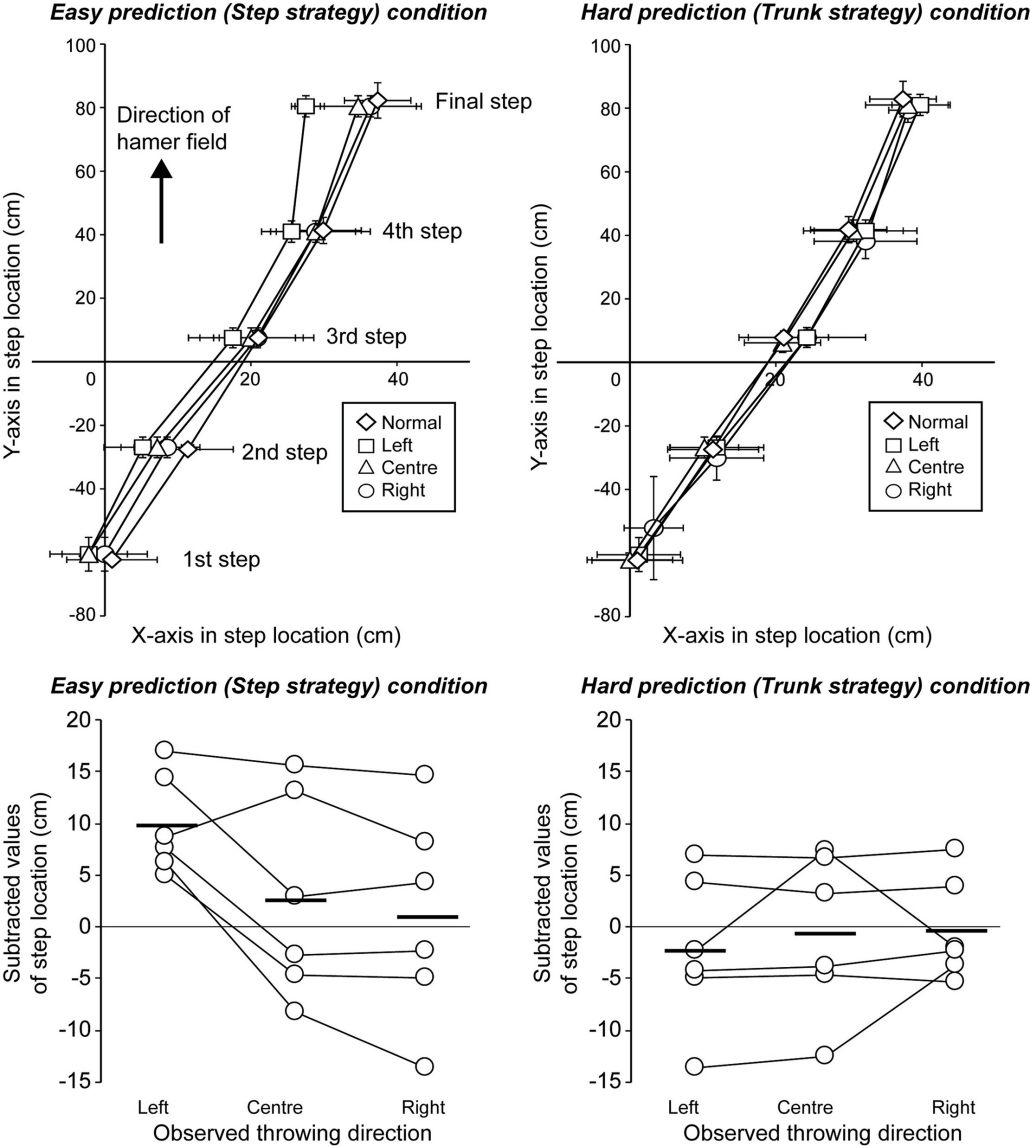
**Actual step location under the EP (left upper panel) and HP (right upper panel) conditions.** The point of origin indicates the center of the hammer throwing circle. The values of the lower panel indicate the individual subtracted values of final step location (X-axis) under each experimental condition (throwing direction and predictability) from the normal condition. Black thick bars indicate average values.

Next, in order to identify the cause of the motor contagion (i.e., whether kinematic imitation was present), we analyzed the movement kinematics of the participants when performing their throws. The upper panels of Fig 4 show the step locations from movement initiation (i.e., initiation of throwing turn movement) to hammer release in each of the EP and HP conditions. The lower panel shows the last step locations in the EP and HP conditions when subtracted from those in the normal throwing condition (see also, S1 Table). If an observer’s own actions were to be altered by those they observed, then in the easy prediction condition (shown in the upper and lower panels on the left of Fig 4) we would expect to see changes in the step location that were significantly greater than those seen in the HP condition (on the right of Fig 4). If the participants were to have fully imitated the kinematics of the model, then the magnitude of the kinematic differences in the EP condition should reflect those demonstrated by the model, as seen in the upper-left panel of Fig 2. The findings revealed that there were indeed kinematic differences between the EP and HP conditions (see Fig 4); this is confirmed by a significant main effect with medium effect size for the predictability of the videos (*F*(1, 5) = 8.33, *p* < .05, η^2^ = .12 [95% CI = .03 - .39], observed power = .64) when comparing the last step in the horizontal (x) axis (lower panel in Fig 4). A significant interaction with medium effect size between direction and predictability (*F*(2, 10) = 4.89, *p* < .05, η^2^ = .08 [95% CI = .01 - .22], observed power = .67) highlighted that the difference between the predictability conditions was largely a result of differences when observing the throws to the left, with subtracted values in the EP condition significantly larger than those in the HP condition (*p* < .01). However, the magnitude of the differences between the EP and HP conditions is clearly small, and markedly less than the kinematic differences observed in the videos (see Fig 2). In addition, there was a significant main effect with small effect size for the direction of the observed throw (*F*(2, 10) = 4.82, *p* < .05, η^2^ = .03 [95% CI = .00 - .10], observed power = .66). This means that although there was a small degree of difference in the kinematics between the EP and HP conditions that was consistent with the changes in the step location seen in the videos, the degree of the differences means that kinematic imitation cannot fully explain the increased motor contagion observed in the EP condition.

**Fig 5:**
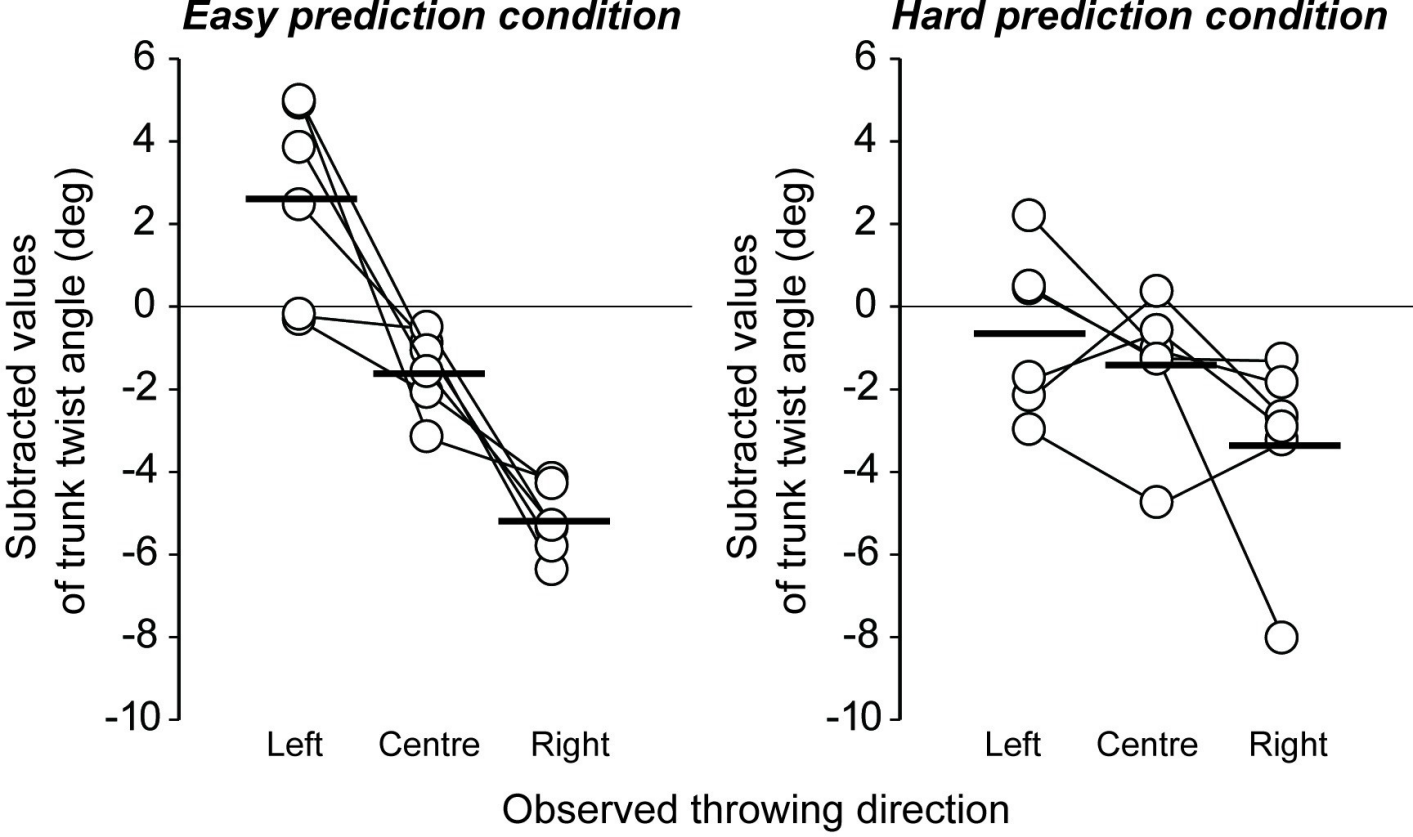
**The subtracted trunk twist angle under the EP (left panel) and HP (right panel) conditions from the normal condition.** Positive values indicate that participants throwing a hammer with a larger twist angle than in the normal condition generally induce the left throwing direction. Negative values indicate the opposite. Black thick bars indicate average values.

Fig 5 shows the participant’s subtracted trunk twist angle at the moment of hammer release in the EP and HP conditions (see also, S1 Table). If a participant’s own actions were to be altered by those observed, then in this figure we should expect to find changes in the trunk angle in the HP condition but not the EP condition, because the throw direction in the HP videos but not EP videos differed on the basis of alterations in the trunk twist angle (see Fig 2). However, in general, the opposite occurred. A significant main effect with large effect size for the direction of the observed throw (*F*(2, 10) = 23.37, *p* < .01, η^2^ = .54 [95% CI = .42 - .69], observed power = .99) and an interaction with medium effect size between the direction of throw and predictability of outcome (*F*(2, 10) = 6.14, *p* < .05, η^2^ = .13 [95% CI = .02 - .29], observed power = .77; main effect of predictability, *F*(1,5) = 0.28, *p* = .62, η^2^ = .00 [95% CI = .00 - .04], observed power = .07) revealed that, contrary to expectations, there were larger changes in the trunk twist angle in the *EP condition* (when viewing videos in which the trunk twist *did not* differ) than there were in the HP condition (when the videos *did* differ in trunk twist). Follow-up testing revealed that, in the EP condition, there were significant differences in the twist angle between each of the comparisons (left-center, left-right, and center-right, *ps* < .05). That is, although the observed model’s trunk twist kinematics in the EP condition were the same irrespective of the direction condition, the participant’s trunk twist angle was more likely to change after viewing the different directions of throw. In sum, trunk angle modulations in participants occurred to some degree in the HP condition, in which the videos were manipulated on the basis of trunk twist angle, but *to a lesser degree* than that recorded in the EP condition, in which the there was no such twist seen in the videos. Our results suggest that motor contagion does not occur on account of kinematic imitation. Changes in throw direction appear to be a result of commensurate alterations in trunk twist angle, irrespective of what was seen in the video.

## Experiment 2

Motor contagion can influence performance in sport both by improving *and* decreasing performance. Therefore, there are situations in which motor contagion is undesirable, yet no studies have made an effort to *inhibit* motor contagion in sports. Rather, several studies have simply proposed that if the motor system *resonates* when observing an action, then an inhibitory mechanism will be required to prevent imitation [27–31]. The common coding explanation for motor contagion assumes that the perception and action systems share common mechanisms for performing and observing the same motor action [19,20]. Therefore, to avoid undesired motor contagion, the shared representation must be controlled when observing the undesirable action. Brass et al. [32] investigated the existence of functional mechanisms and brain circuits that are involved in the control of shared representations. They reported that when there was a reduction in the imitative response in a motor contagion task [9], the medial prefrontal cortex yielded higher activation values [27, 33]. Furthermore, they reported that overlapping brain activation could be found in the anterior fronto-medial cortex and the temporo-parietal junction area for the control of shared representations when performing self-related processing tasks such as self-referential and mental state attributions.

Given the potential overlap with self-related processing tasks, Spengler et al. [30] investigated whether self-focus led to a decrease in motor contagion. In their study, self-focus was elicited by engaging participants in a self-referential task before action observation, namely, judging evaluative statements (e.g., ‘‘Leipzig is a pleasant town”) that involved the person’s value system. As a result, motor contagion was reduced. Moreover, Schütz-Bosbach et al. [29] reported that if an observed action was attributed to the participants themselves, the activation of the motor system involved in shared representation was reduced; if an observed action was attributed to another agent, the activation of the motor system involved was enhanced. These findings indicate that self-processing enhances one’s own action control mechanisms. This is because it inhibits the activation of shared representations needed to resist unintentional motor contagion. Therefore, the purpose of this second experiment was to verify whether motor contagion could be inhibited by self-focus during the observation of others’ actions (Experiment 2).

To accomplish this purpose, we manipulated the direction of focus by using self-talk during action observation. Self-talk involves statements that are addressed to oneself and not to others [34]; it is useful in offering instruction on how to achieve a goal [35]. Self-talk is achieved using inner speech that is self-directed and/or self-referential [36]. Thus, we hypothesized that it would lead participants to focus on self-related processing. According to previous evidence, self-focus leads to a greater ability to execute one’s own motor intention and to resist the effect of motor contagion [30]. Therefore, we hypothesized that motor contagion would decrease when participants participated in self-talk with self-focus during action observation. If true, it could provide a useful method to reduce unwanted contagion in sports.

### Methods

#### Participants

The same participants from Experiment 1 took part in Experiment 2.

#### Apparatus and stimuli

In Experiment 2, we used the same apparatus and stimuli used in the EP condition in Experiment 1, because motor contagion was greater in this condition than it was in the HP condition. In this experiment, we did not capture motion kinematics.

#### Procedure

Experiment 2 was conducted on a different day from Experiment 1. The procedure was identical to Experiment 1, except that in Experiment 2, participants were required to throw a hammer both in a self-focus condition and in a non-self-focus condition. In order to manipulate self-referential processing as a dependent variable, the participants were required to mutter under their breath about either their own performance (*self-focus*) or about another’s performance (*non-self-focus*) during action observation prior to throwing the hammer themselves. In the self-focus condition, participants were instructed to say they would ‘do my best movement in the next trial’ while watching videos, but were reminded to pay attention to the model’s movement in order to prevent reduced attention to action observation. We expected that the self-talk, indicated by the phrase ‘do my best movement in the next trials,’ would induce self-referential processing. On the other hand, in the non-self-focus condition, participants were instructed to say ‘(you) make mistakes’ (referring to the model) while watching videos and to pay attention to the model’s movements. We expected that muttering under their breath about another’s performance (i.e., the model’s performance) would induce less self-referential processing in the non-self-focus condition than self-talk did in self-focus condition. The hammer was thrown 15 times (5 times for each direction stimuli) for each condition (30 throws in total). Participants were instructed to throw the hammer toward the center of the field, irrespective of the observed stimuli. The order of direction stimuli was randomized, and the order of the self- and non-self-focus conditions was counterbalanced across participants.

#### Measurements and data analysis

To clarify whether self-focus while observing others’ actions was effective to inhibit motor contagion, we calculated the direction of throw for the self- and non-self-focus conditions and subtracted each from the direction of the normal throws recorded during Experiment 1. Subsequently, the means of the subtracted throwing direction were analyzed using a 3 (Direction: left, center, and right) × 2 (Self-focus: self-focus and non-self-focus) ANOVA. Effect sizes were reported as η_p_^2^, and post-hoc mean comparisons were performed using Bonferroni corrected t-tests.

### Results and discussion

#### The impact of self-focus on motor contagion

**Fig 6:**
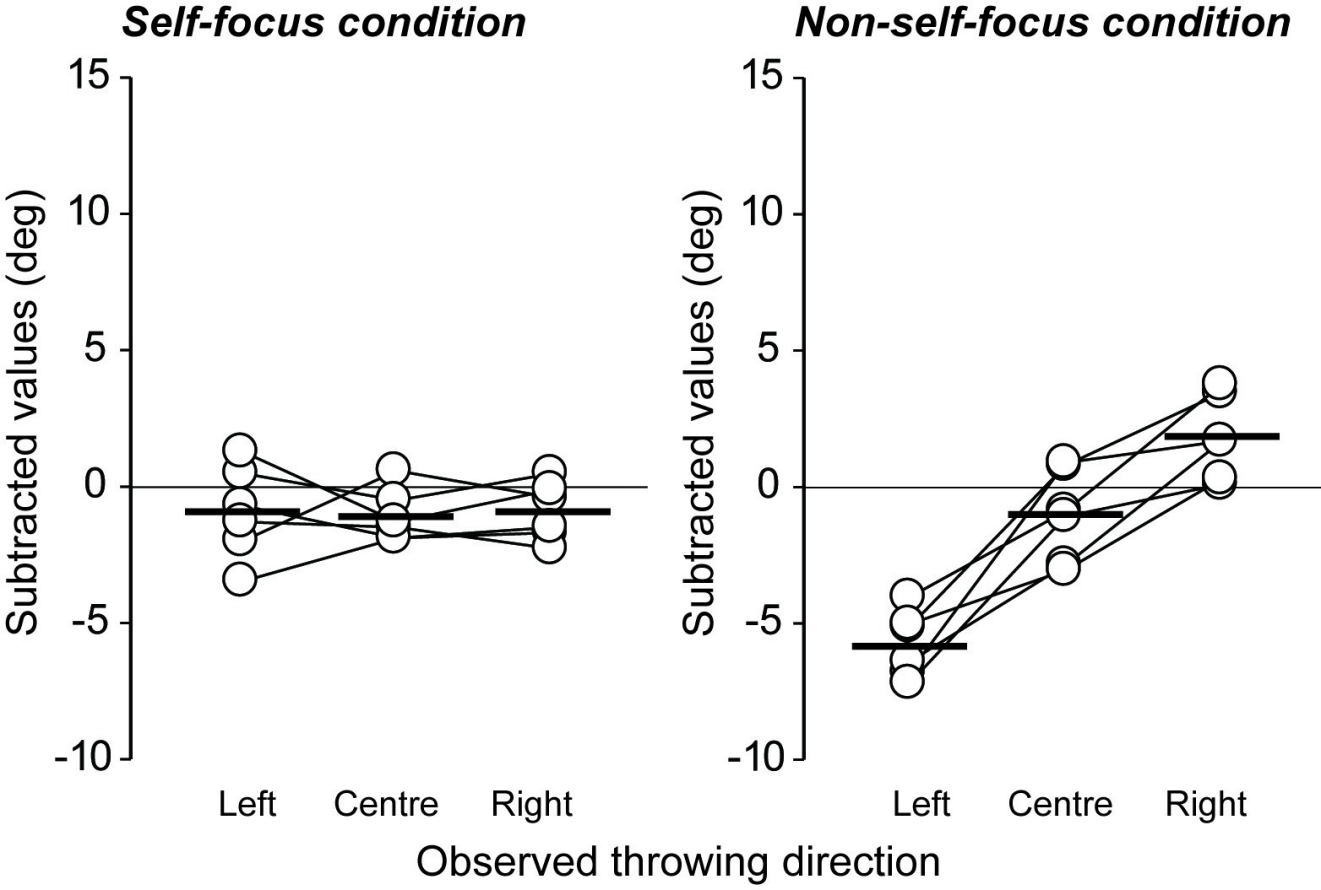
**Individual data of hammer throwing direction under the self- (left panel) and non-self-focus (right panel) conditions for each observed throwing direction.** The subtracted values (y-axis) indicate the change in throwing angle for each throwing condition (with video observations) when compared to the normal throwing condition (without video observation). In the self-focus condition, participants were required to focus on their own throwing during observation of the model’s kinematics. In the non-self-focus condition, they fully focused on the model’s throwing. Black thick bars indicate average values.

Fig 6 shows the direction of throw in the self- and non-self-focus conditions (see also, S1 Table). A significant main effect with large effect size for the direction of the observed throw (*F*(2, 10) = 25.69, *p* < .01, η^2^ = .37 [95% CI = .23 - .49], observed power = .99) and a direction × self-focus interaction with large effect size (*F*(2, 10) = 53.35, *p* < .01, η^2^ = .37 [95% CI = .24 - .47], observed power = .99; main effect of predictability, *F*(1,5) = 2.96, *p* = .15, η^2^ = .02 [95% CI = .01 - .11], observed power = .29) revealed a significant effect of motor contagion in the non-self-focus condition, but none in the self-focus condition. Specifically, in the non-self-focus condition there was a significant difference in the subtracted direction of throw when observing videos throwing to the left and center, left and right, and center and right (*p* < .01). That is, we replicated the motor contagion phenomena observed in Experiment 1 despite the addition of the instruction. In contrast, there was no significant difference between any of the throw directions in the self-focus condition. This means that the contagion phenomenon disappeared, consistent with the study of Spengler et al. [23] that reported an inhibitory effect of self-focus on automatic imitation. Therefore, our data supports the idea that that motor contagion can be eliminated using a self-focus approach.

## General discussion

In sports settings, athletes and coaches often talk about contagion, in which they try to “go with the flow” after good performances or to “stem the tide” after bad performances of teammates. Given the potential impact of contagion in sport, the aims of this study were to identify both (a) whether the ability to predict the outcome of an observed action would influence the degree of motor contagion (Experiment 1), and (b) whether motor contagion could be inhibited by self-focus during the observation of others’ actions (Experiment 2). While previous research on motor contagion in sports presented participants with only action *outcome* stimuli [7], in this study we presented action kinematics that were linked to specific outcomes. In addition, two different types of action stimuli (HP and EP) were employed to manipulate the degree to which the action outcome could be predicted on the basis of the model’s kinematics, in order to clarify whether motor contagion is underpinned by the observer’s ability to associate the action with the action outcome. Indeed, motor contagion was found to be greater when observing a more predictable model’s actions (Fig 3), suggesting that the link between the observed kinematics and the outcome is crucial in generating the contagion. This prediction dependency effect is consistent with the evidence that experts experience greater motor contagion when they increase their ability to predict the outcome of movement kinematics [3].

Motor contagion is often explained by common coding theory, which assumes that common representations underpin the execution and observation of motor actions [19,20]. According to this theory, a perceived action automatically induces a motor representation of the observed action in the perceiver’s motor system. That is, the perceived actions of another easily influence an observer’s action. This account has been further supported by the existence of mirror neurons in the motor system [37,38] that are activated both when observing others and when performing the same action oneself [38,39]. The motor system is activated when an observed action is goal-directed, even if a part of the action is invisible [21]. Furthermore, expert athletes with superior outcome prediction abilities [40–42] activate the motor system when they perform outcome prediction tasks [22–25], an activation that might be related to successful prediction because the motor system is activated more in successful prediction than it is in unsuccessful prediction [26]. Accordingly, if the common code mechanism were to be associated with motor contagion, then shared representations in participants should have been more active in the EP condition in which action prediction was easier (success more than 80%), compared with the HP condition in which prediction was much harder (less than 50%). That is, because outcome prediction is likely to simulate internally and/or resonate with perceived events using one’s own motor system (for review, [21]), then motor contagion should be expected to be greater after observing actions where the outcome is more apparent to the observer.

We analyzed each participant’s motion kinematics during throwing to identify the nature of the motor contagion that occurred after observing others’ actions. Motion analysis revealed that while some changes in kinematics were apparent, those changes were not consistent with what would be expected as a result of kinematic imitation (Figs 4 and 5). In particular, participants modulated their trunk twist angle unintentionally (because they intended to throw in the center direction in all conditions), even when presented with stimuli that were manipulated only in step location and not in their trunk twist angle (see Fig 2). Therefore, our results indicate that motor contagion after observing others’ actions is not caused by pure kinematic imitation of the observed action; rather, it may be the imitation of the *outcome* of the model’s action. Furthermore, because of our instruction to throw towards the center of the field, irrespective of what the participant observed when watching the model, it appears as though motor contagion was induced automatically [12].

It has been reported that action observation promotes (or interferes with) the performance of an action that is similar, not in its body movement topography, but in its effects on an environmental object. For example, Edwards et al. [13] asked participants to observe others performing a goal-directed action (reach and grasp) toward either a large or small object, with participants subsequently performing the same action for an object of the same size (congruent condition) or a different size (incongruent condition). The participants displayed a shorter time to peak velocity in their own actions in the congruent condition than they did in the incongruent condition, implying that the congruent observed action implicitly affected the participant’s own action. In another condition, the participants did not observe the reaching action but observed only the object. Interestingly, even in the absence of an action, the time to peak velocity in the participant’s own actions was again reduced during the congruent (object observation) condition compared to the incongruent condition. This result indicates that an object representation that codes the goal of an observed action may activate motor representations that lead to the achievement of the same goal. That is, this evidence suggests that motor contagion in our study may have been caused by an increased understanding the observed action goal and/or outcome. Therefore, participants did not imitate the model’s *kinematics* in the EP task; rather, they unintentionally modulated their trunk twist angle (and their foot placement to a much lesser degree) to achieve an estimated outcome according to the inferred intention/goal from the action kinematics observed.

An understanding of why motor contagion occurs in sports will not only further our theoretical understanding of the phenomenon; it will also shed light on how to alleviate unwanted performance decrements. As noted, Spengler et al. [30] demonstrated that self-focus activates the anterior front-medial cortex (aFMC), and that this region is also activated when imitation must be inhibited [32]. Taken together, these findings suggest that humans have a system that is capable of enhancing their own action control and inhibiting motor contagion. In Experiment 2, according to Spengler’s [30] study, we manipulated the direction of focus during action observation and found that motor contagion could be inhibited through self-talk that enhanced self-related processing. Our results suggest that experimental laboratory findings can be successfully applied in sports settings to reduce contagion. Recently, some laboratory studies have shown that motor contagion can be controlled (inhibited or facilitated) by manipulating the imitation intention (whether an imitative or a counterimitative response [43]) and motor imagery during observation [44]. In addition, the human brain controls behavior optimally via a fine interplay between motor facilitation and inhibition; this is accomplished by means of the corticocortical connectivity between the dorsal premotor cortex and the primary motor cortex [45,46]. Based on the notion of motor simulation, we might use the same motor inhibitory process irrespective of self–other actions. Further investigation is needed to identify how to control the motor contagion effect to maximize sport performance.

A particular strength of our study is that we have been able to demonstrate motor contagion in highly skilled (national level) athletes, though the expertise level of our participants meant that we had only a limited sample of participants available from which to test. The decision to test highly skilled athletes was motivated by previous findings which have shown that contagion increases in line with an increase in the expertise level of the participants [7]. And given that the ability to anticipate the outcome of an opponent’s action increases commensurate with the skill level of the athlete [40], the recruitment of expert athletes allowed us in Experiment 1 to best test the influence of the link between observed kinematics and outcome in generating contagion. Of course, though, the limited sample size resulting from the availability of expert athletes does increase the risk of a Type 1 error in our experiments. The clear statistical findings, along with the large effect sizes, do provide some reassurance about the veracity of the findings. Moreover, the individual participant data shown within the plots tells in most parts a clear story that the manipulations had systematic effects on most if not all of the participants. Nonetheless, it is important for future work to demonstrate not only the replicability of the findings, but to test how widely they might generalize to participants of lesser skill in the task in question.

From these two experiments, we concluded that the link between observed kinematics and outcome is crucial in generating contagion. In addition, the cause of the contagion phenomenon is the automatic imitation of an action outcome induced by predictable action observation. Further, motor contagion may be inhibited through the self-focus that enhances self-related processing. The present study, however, did not assess whether such self-talk actually induced self-referential processing. Therefore, to clarify the cause of motor contagion inhibition, further experiments with neuroscientific methods such as transcranial magnetic stimulation (TMS) [29] are warranted. Moreover, although this is the first study to focus on the inhibition of motor contagion in sport, we only focused on the observation phase. In sport settings, athletes do not know whether a positive or negative event will occur during observation. Therefore, athletes must counteract a negative contagion effect after having observed an undesirable action. Relatedly, Gray and Beilock [7] demonstrated that as the delay increased between the inducing stimulus and action execution, the magnitude of the contagion effect decreased. Therefore, if there is a risk of negative contagion, it may be effective for athletes and coaches to take a break, such as a time-out. In contrast, if athletes observe a successful result by others, they should act immediately to take advantage of the momentum provided by the previous action. In fact, recently, Ubaldi and colleagues [31] found the mechanisms by which the brain is both tuned to produce imitative responses in a fast-automatic way and also capable of overriding them by means of a parallel, more flexible visuomotor coupling that follows arbitrary visuomotor associations. Further ideas for counteracting motor contagion after action observation are needed based on such a mechanism.

## Supporting information

S1 Table The individual data of outcome and kinematic parameters of hammer throwing in Experiment 1 and 2. (XLS)
